# Supplementary material for: The Glycoprotein M6a Is Associated with Invasiveness and Radioresistance of Glioblastoma Stem Cells
Source: Cells. 2022 Jul 6;11(14):2128. doi: 10.3390/cells11142128 (PMC9321762; doi:10.3390/cells11142128)
Supplement: Supplementary file 1 [file cells-11-02128-s001.zip › Figure S1.pdf]

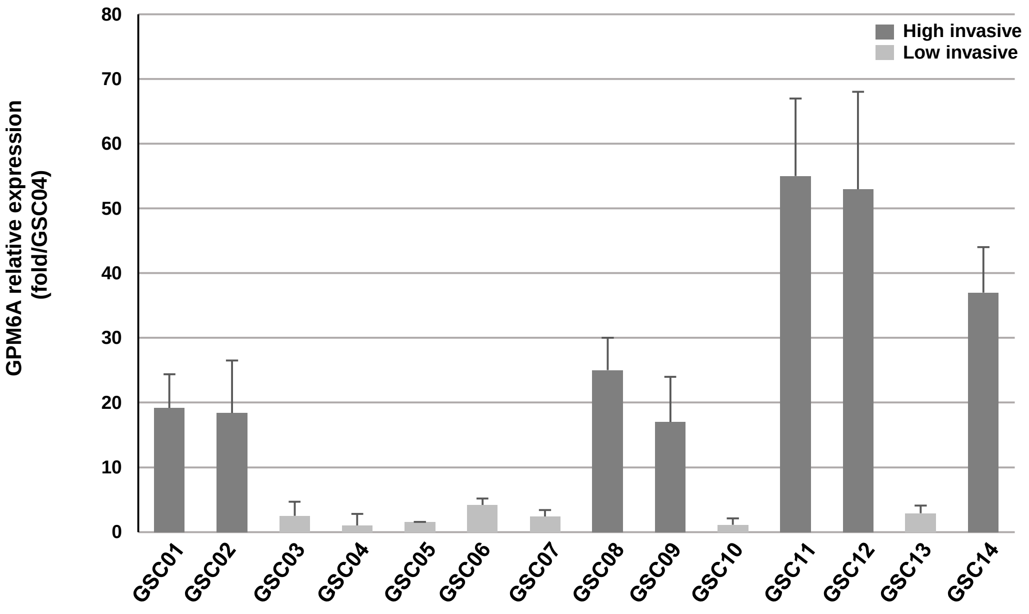

Figure S1. GPM6A is over-expressed in the highly invasive GBSC. mRNA expression of GPM6A was analyzed by real time PCR in the 14 primary cultures of GBSC isolated from 14 patient samples. GAPDH was used for normalization. Results are presented as fold compared to GSC04 expression (which presents the lowest expression). Quantifications of 3 experiments are presented as means ± SD.
