# Supplementary material for: The Glycoprotein M6a Is Associated with Invasiveness and Radioresistance of Glioblastoma Stem Cells
Source: Cells. 2022 Jul 6;11(14):2128. doi: 10.3390/cells11142128 (PMC9321762; doi:10.3390/cells11142128)
Supplement: Supplementary file 1 [file cells-11-02128-s001.zip › Figure S2.pdf]

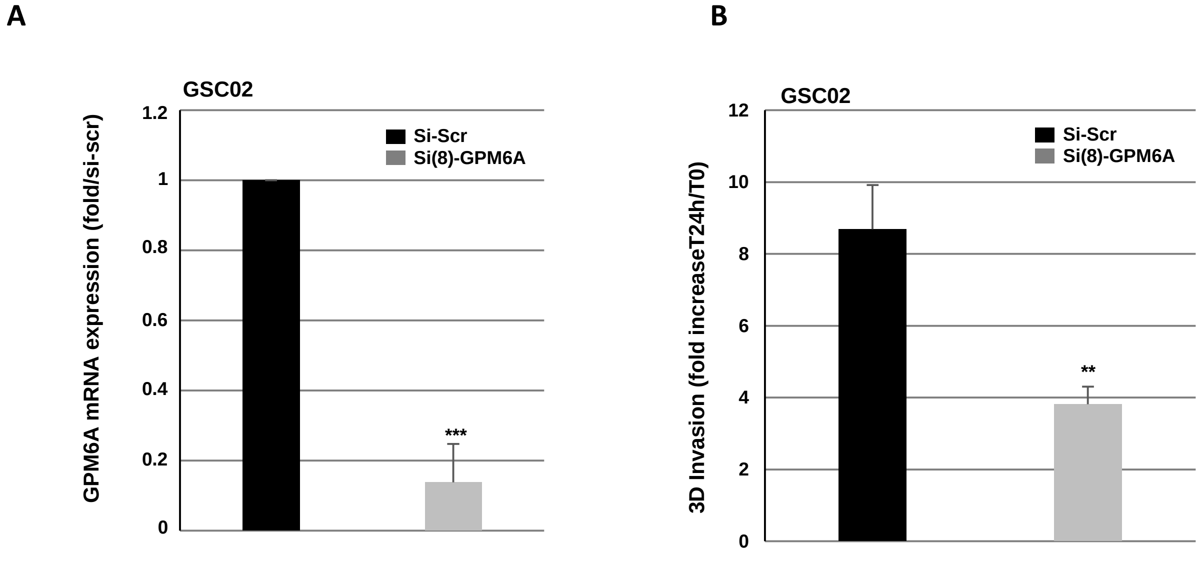

Figure S2. Checking of a potential off target effect with GPM6A siRNA. (A, B) GSC02 primary neurospheres were transfected with a specific siRNA (si(8)-GPM6A or a scramble control (si-Scr) as indicated. (A) GPM6A mRNA expression was analyzed by real time PCR. (B) Quantification of 3 D tumor cells invasion was performed with GC02 primary neurospheres as described in « Methods ». Quantifications of 3 experiments are presented as means SD. ***P<0.001; **0.001<P<0.01.
