# Supplementary material for: The Glycoprotein M6a Is Associated with Invasiveness and Radioresistance of Glioblastoma Stem Cells
Source: Cells. 2022 Jul 6;11(14):2128. doi: 10.3390/cells11142128 (PMC9321762; doi:10.3390/cells11142128)
Supplement: Supplementary file 1 [file cells-11-02128-s001.zip › Table S1.pdf]

GBSC	Spheres formation In vitro	Differentiation In vitro	Tumor In vivo	Subtype	GBSC markers expression (SOX2, Olig2)
GSC01	+	+	+	PN	yes
GSC02	+	+	+	PN	yes
GSC03	+	+	+	PN	yes
GSC04	+	+	+	PN	yes
GSC05	+	+	+	PN	yes
GSC06	+	+	+	PN	yes
GSC07	+	+	+	PN	yes
GSC08	+	+	+	MES	yes
GSC09	+	+	+	PN	yes
GSC10	+	+	+	MES	yes
GSC11	+	+	+	PN	yes
GSC12	+	+	+	PN	yes
GSC13	+	+	+	PN	yes
GSC14	+	+	+	MES	yes

Table S1: GBSC characterization. GBSC isolated from patient specimen were characterized for their ability of self-renewal by sphere formation assays in vitro, their pluripotent aptitude to differentiate into neural lineages in vitro (Differentiation), their high tumorigenic potential in vivo by orthotopic xenograft in nude mice (Tumor in vivo) and the overexpression of stem cell markers (SOX2, OLIG2). The subtype proneural (PN) or mesenchymal (MES) was also determined.
